# Coverage‐Controlled Superstructures of *C*_3_‐Symmetric Molecules: Honeycomb versus Hexagonal Tiling

**DOI:** 10.1002/anie.202001383

**Published:** 2020-03-20

**Authors:** Torben Jasper‐Tönnies, Manuel Gruber, Sandra Ulrich, Rainer Herges, Richard Berndt

**Affiliations:** ^1^ Institut für Experimentelle und Angewandte Physik Christian-Albrechts-Universität 24098 Kiel Germany; ^2^ Otto-Diels-Institut für Organische Chemie Christian-Albrechts-Universität 24098 Kiel Germany

**Keywords:** *C*_3_-symmetric molecules, coverage control, honeycomb superstructure, molecular tiling model, scanning tunneling microscopy

## Abstract

The competition between honeycomb and hexagonal tiling of molecular units can lead to large honeycomb superstructures on surfaces. Such superstructures exhibit pores that may be used as 2D templates for functional guest molecules. Honeycomb superstructures of molecules that comprise a C_3_ symmetric platform on Au(111) and Ag(111) surfaces are presented. The superstructures cover nearly mesoscopic areas with unit cells containing up to 3000 molecules, more than an order of magnitude larger than previously reported. The unit cell size may be controlled by the coverage. A fairly general model was developed to describe the energetics of honeycomb superstructures built from *C*_3_ symmetric units. Based on three parameters that characterize two competing bonding arrangements, the model is consistent with the present experimental data and also reproduces various published results. The model identifies the relevant driving force, mostly related to geometric aspects, of the pattern formation.

## Graphical abstract

**Molecular honeycomb** superstructures were investigated using scanning tunneling microscopy, covering nearly mesoscopic areas and with unit cells containing up to 3000 molecules. A fairly general model was developed that describes the energetics of such structures and show that their sizes can be controlled by coverage.

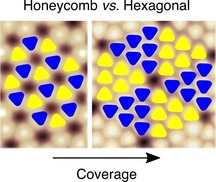

## Introduction

Molecular self‐assembly on substrates may be used to fabricate desired nanostructures on surfaces. The assembly process is initiated and controlled by the molecule–substrate and molecule–molecule interactions. The former interaction ideally ensures the stable adsorption of the molecules and their efficient diffusion on the surface at suitable temperatures.^1^ The molecule–molecule interactions usually determine the self‐assembled molecular patterns. Often weak interactions are used such as hydrogen bonding, dispersion forces, π–π stacking, metal coordination, and electrostatic interactions.^2^, ^3^, ^4^, ^5^ Local, directional, and selective molecule–molecule interactions, for example, hydrogen bonding and metal coordination, are particularly attractive because they enable further control of the patterns via suitable design of molecules.^6^

A vast variety of molecular surface tilings, both periodic and nonperiodic, have been reported.^1^, ^5^, ^7^, ^8^, ^9^, ^10^, ^11^, ^12^, ^13^, ^14^, ^15^ In particular, a competition between honeycomb and hexagonal arrangements of *C*_3_‐symmetric molecules can lead to honeycomb superstructures, which have attracted considerable interest for several reasons. These superstructures exhibit cavities that may serve to arrange functional guest molecules or for synthetic molecular recognition.^1^, ^16^, ^17^, ^18^, ^19^, ^20^, ^21^, ^22^ Furthermore, a variety of periodic patterns exhibiting different pore‐to‐pore distances have been obtained employing a single compound on a given surface.^23^, ^24^, ^25^, ^26^, ^27^, ^28^, ^29^, ^30^, ^31^ It has been reported that the number of molecules composing the unit cells is affected by the molecular coverage.^26^ Although this control via coverage should, in principle, enable superstructures of any size, the largest unit cells reported so far were comprised of some two hundred molecules per unit cell. Furthermore, honeycomb superstructures may turn out useful to control the density of functional molecules on surfaces. In the context of platform molecules,^32^, ^33^, ^34^ where a functional unit is attached to a molecular base,^35^, ^36^, ^37^, ^38^, ^39^, ^40^, ^41^, ^42^ the pattern of the platforms is imposed on the functional units.

Herein, we report on coverage‐controlled molecular superstructures of a *C*_3_‐symmetric molecule on Ag(111). While the molecule has lateral dimensions of about 1 nm, the superstructures have lattice parameters exceeding 50 nm, contain up to approximately 3000 molecules per unit cell, and cover nearly mesoscopic surface areas. The present molecular unit is a platform onto which different functional groups can be attached.^43^, ^44^, ^45^ Moreover, we developed a model describing the dimension of the honeycomb superstructures of *C*_3_‐symmetric molecules. According to the model, the geometric properties of the superstructures essentially depend on three parameters related to the two competing molecule–molecule interactions that favor either hexagonal and or honeycomb arrangements. The model explains the large superstructures reported here and also reproduces previously observed superstructures of various *C*_3_ molecular units. The parameters of the model can in principle be inferred from force‐field calculations with moderate computational effort. Therefore, it may be employed to predict geometric properties of new molecules and to guide the design of new *C*_3_ molecules to realize particular honeycomb superstructures.

## Results and Discussion

### Preliminary Results for Methyl Trioxatriangulenium

**Figure 1:**
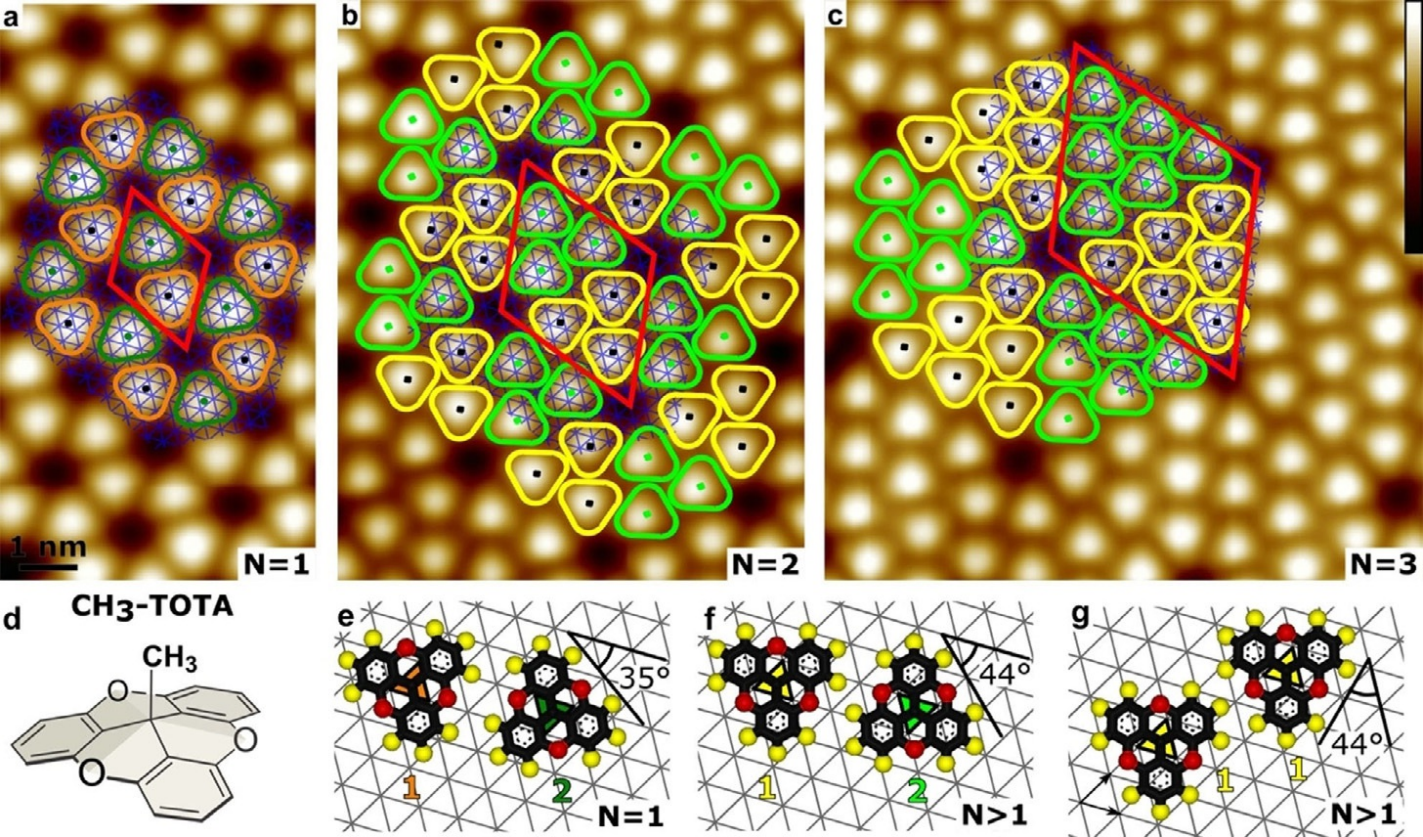
a)–c) Constant‐current STM topographs of a series of honeycomb superstructures of Me‐TOTA on Au(111) (tunneling parameters: 30 pA, a) 1 V, b),c) 100 mV). Some molecules are marked with rounded triangles; the color is representative of the molecular adsorption site (hollow site in *hcp* or *fcc* position of the substrate lattice) and of the orientation of the molecule relative to the substrate (see (e)–(g)). The vertices of the blue hexagonal meshes indicate the approximate positions of the underlying surface Au atoms neglecting the herringbone reconstruction. Red rhombi show the unit cells of honeycomb superstructures of orders a) *N*=1, b) *N*=2, and c) *N*=3. The color scale shown as an inset in (c) ranges over 0.23 nm and is common for the three topographs. d) Lewis structures of methyl trioxatriangulenium (Me‐TOTA). e)–g) Pairwise configurations commonly observed for Me‐TOTA on Au(111). Scaled gas‐phase models of Me‐TOTA are overlayed on hexagonal meshes indicating the Au(111) surface atoms (nearest neighbor distance *a=*0.288 nm). Hydrogen (oxygen) atoms are depicted by yellow (red) spheres. The center‐to‐center distances between the molecules are $\sqrt{37/3}a$ =1.01 nm for (e) and (f) and $\sqrt{13}a$ =1.04 nm for (g).

For the experiments we used the compound methyl‐trioxatriangulenium (Me‐TOTA, Figure 1 d) for a number of reasons. This molecule may be sublimated clean and intact in an ultra‐high vacuum environment, which enables convenient control of the surface coverage.^43^, ^44^, ^45^ Its *C*_3_ symmetry allows for a range of molecular assemblies. The molecule is mobile on the surface when prepared at suitable temperatures, which is essential for the molecules to be able to explore different superstructures. Furthermore, the TOTA platform and the related compound triazatriangulenium are very versatile and the methyl moiety may be exchanged for other moieties of interest. This has been demonstrated for small moieties such as hydrogen, ethyl, ethynyl, and propynyl^43^, ^44^, ^45^ as well as porphyrins, diazocine, norbornadiene, imine, and azobenzene derivatives.^32^, ^35^, ^40^, ^41^, ^46^, ^47^, ^48^

Using low‐temperature scanning tunneling microscopy (STM) along with density functional theory (DFT) calculations^43^, ^44^, ^45^ we previously showed that the TOTA platform lies flat on Au(111) substrates with the attached moiety standing vertical (Figure 1 d). Me‐TOTA binds to Au(111) via physisorption with an adsorption energy on the order of −2 eV, which is comparable to that of a covalent bond. This large binding energy is caused by the extended π‐electron system of the platform. The adsorption is strongest when the center of the molecule is located above a hollow site of the Au(111) surface.

Below we first present the patterns formed by Me‐TOTA on Au(111). While fairly large superstructures were observed, we suspected that the herringbone reconstruction of this substrate may be a limiting factor for the self‐assembly process and therefore extended our study to Ag(111). Indeed, much larger superstructures were achieved as presented below.

### Experimental Results on Au(111) Substrates

At low coverages, Me‐TOTA forms a honeycomb mesh on Au(111) (Figure 1 a). A unit cell with two molecules is indicated by a red rhombus whose corners are located at pores of the molecular network. We label the structures by the number N of molecules along the line connecting two adjacent pores. According to this definition, Figure 1 a shows a *N*=1 superstructure.

Figure 1 e displays a model of a pair Me‐TOTA molecules that is based on the STM observations. The two molecules are rotated by 60° with respect to each other, which enables the formation of two O⋅⋅⋅H hydrogen bonds. The sides of the molecules form an angle of 35° with a densely packed direction of the Au substrate (Figure 1 e) rendering the adsorption geometry chiral. For an isolated molecule, DFT calculations predict a very similar value of 36°.^44^

Furthermore, the pairwise interactions make the honeycomb structures chiral as well. For example, the O atom of the left molecule in Figure 1 e binds to the H atom located below the O atom of the right molecule. In the other enantiomer (Supporting Information, Section II) the H atom above the O atom of the right molecule is involved in bonding.

The structure of Figure 1 e involves the occupation of two hollow sites (marked green and orange for the left and right molecule, respectively) that correspond to *hcp* and *fcc* positions of the Au lattice. The calculated energy difference between these sites (≲30 meV) is within the uncertainty of DFT calculations.^45^

On sample areas with different local molecular densities, other ordered superstructures were observed (Supporting Information, Section II). While they exhibit the same symmetry as the simple honeycomb pattern the sizes of the unit cells are larger. Figures 1 a–c show examples of *N*=1, 2, and 3 superstructures. The number of molecules per unit cell (red rhombi) is *N_N_*=*N*(*N*+1), that is, 2, 6, and 12 molecules, respectively. Each unit cell is comprised of two subunits with hexagonal packing of the molecules that corresponds to a $\sqrt{13}\times \sqrt{13}R:13.9^{\circ }$ mesh relative to the underlying Au plane. The equivalent matrix notation of the structure reads $\left(\begin{array}{cc} 3 & 1\\ -1 & 4 \end{array}\right)$ . The subunits are different in terms of the molecular orientations (rotated by 60°) and the adsorption sites (*fcc* vs. *hcp*). The molecules are arranged in a corner‐to‐side manner within the subunits (for example, molecules marked in yellow in Figure 1 c), and side‐by‐side at the subunit boundaries. Closer inspection of the side‐by‐side arrangement (Figure 1 f) reveals a subtle difference of the *N*>1 structures compared to the simple *N*=1 honeycomb mesh. The angle between a densely packed direction of the substrate and the side of a molecules is 44° rather than 35°. This small rotation leads to corner‐to‐side orientation that improves O⋅⋅⋅H bonding (Figure 1 g). Neighbors share one such bond in the subunits whereas two hydrogen bonds occur in the side‐by‐side configuration at boundaries. Naively it may be expected that the molecules will form patterns that maximize the number of double hydrogen bonds. However, as will be shown below, this is not the primary driving force. We observed Me‐TOTA superstructures up to *N*≈8 on Au(111). The structures have an epitaxial relation with the underlying surface within the uncertainty of the calibration of the piezo scanner of <5 %. To the best of our knowledge, such a relation has not been reported before for honeycomb superstructures.^23^, ^24^, ^25^, ^26^, ^27^, ^28^, ^29^, ^30^, ^31^ We therefore hinted that the herringbone reconstruction of Au(111) may prevent the formation of superstructures with larger *N* (Supporting Information, Section II). To test this hypothesis, we used a Ag(111) substrate. Its lattice parameter is very close to that of Au(111) and its surface is unreconstructed and regular over large terraces.

### Large Honeycomb Superstructures on Ag(111)

**Figure 2:**
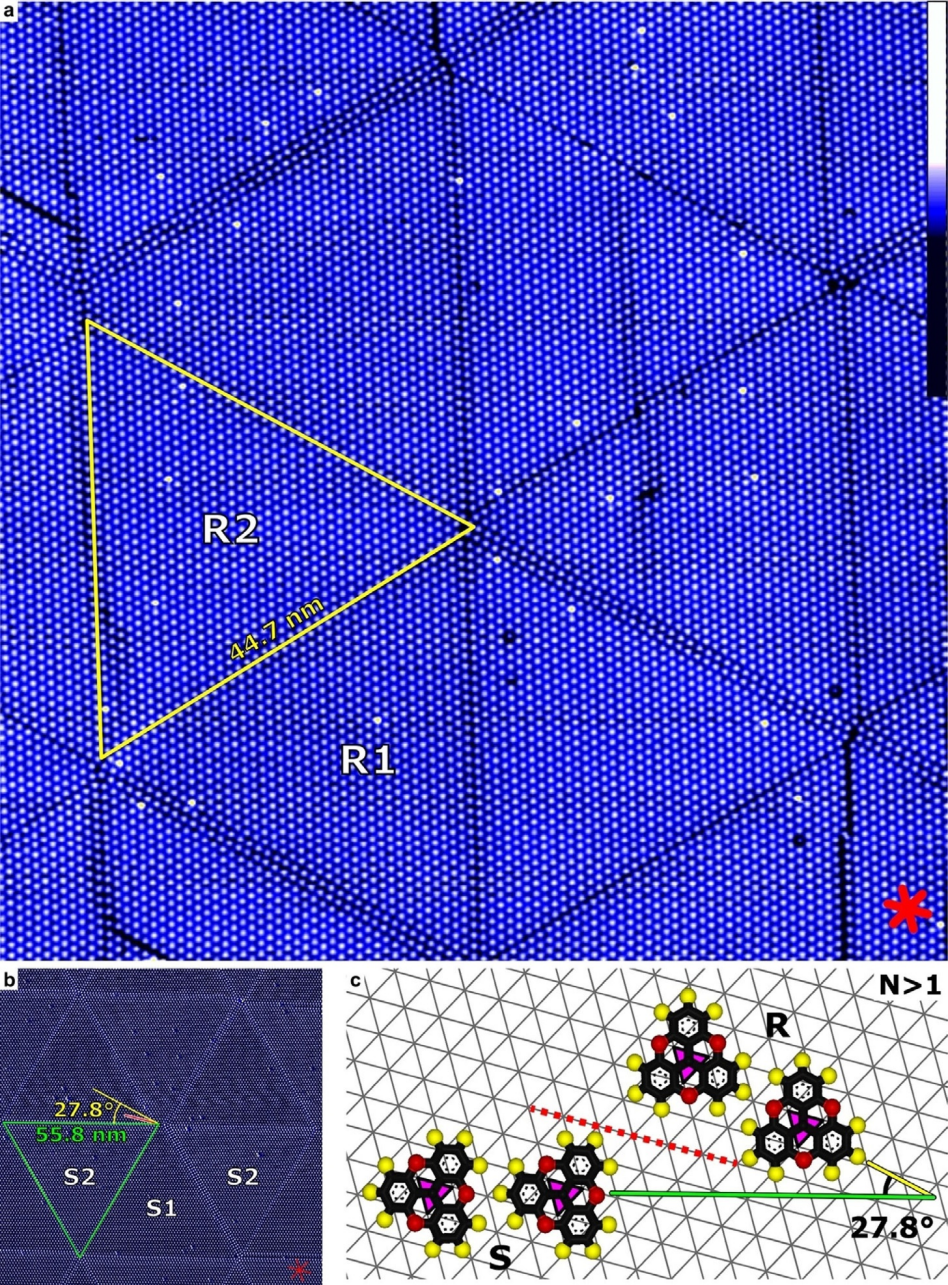
a) Constant‐current STM topograph of a *N*=43 superstructure of Me‐TOTA on Ag(111) (Image width: 97 nm; tunneling parameters: 1 V, 30 pA). Each bright dot corresponds to a single molecule. Triangular areas (example indicated by a yellow triangle) corresponding to half of the superstructure unit cell exhibit edges with 43 molecules (neglecting defects). Red lines (lower‐right corner) indicate densely packed directions of the Ag(111) surface. The brightest spots may be due to ethyl TOTA impurities (on a per mil level). Inset on the right: false‐color scale. b) STM image of a *N*=54 Me‐TOTA pattern on Ag(111) with the opposite chirality as reflected by the domain orientations in (a) (yellow) and (b) (green). Each unit cell contains almost 3000 molecules. The data shown are the deviations (±10 %) of the tunneling current from a constant value of 23 pA at 0.8 V. c) Corner‐to‐side molecular arrangements relative to the silver‐atom mesh (gray) for the enantiomers S and R. The yellow and green lines indicate the orientation defined by the corner‐to‐side stacking of the molecules in the domain R and S, respectively. The dashed red line is oriented along a densely packed atomic row. The letters in (b) and (c) specify the enantiomers in the corresponding domain, while the numbers indicate adsorption sites (1 and 2 for *fcc* and *hcp* hollow sites).

The deposition of Me‐TOTA on Ag(111) at ambient temperature produces very large honeycomb superstructures. Figure 2 a shows a *N*=43 mesh. The distance between pores is 44.7 nm and each unit cell comprises about 1900 molecules. Another example of a large superstructure with *N*=54 corresponding to approximately 3000 molecules per unit cell is shown in Figure 2 b. Interestingly, the lines separating unit cells exhibit different orientations than those of Figure 2 a (compare, for instance, the yellow and green triangles in Figure 2 a,b, respectively). A more detailed analysis shows that a chirality is induced by adsorption of the molecules on the Ag(111) mesh. The corner‐to‐side arrangement of the molecules, occurring at the borders of hexagonal domains (for example, triangles in Figure 2 a,b), defines a direction relative to the underlying substrate. The molecules in domains R and S (green and yellow triangles in Figure 2 a,b) arrange themselves along axes (green and yellow lines in Figure 2 c) that are rotated by ±13.9° relative to a densely packed atomic row (dashed red line in Figure 2 c). In other words, R and S are rotational domains (27.8° rotation).

A detailed analysis reveals that the pairwise interactions and hence the honeycomb structures observed on Ag(111) are essentially the same as on Au(111) (Supporting Information, Section III).

### Model

To interpret the evolution of the unit cells from 2 molecules in the *N*=1 honeycomb structure to huge cells with *N*=54 we developed a model that considers *C*_3_ symmetric molecules with two interactions that favor either honeycomb or hexagonal patterns. Related models have been previously reported for specific systems. Ye et al.^26^ assumed that trimesic acid molecules maximize the density of double hydrogen bonds, which leads to a coverage dependence of *N*. Xiao et al.^27^ considered the intermolecular interaction energy per surface area as a function of *N*. Both models invoke energy density rather than total energy. However, for a given coverage, one would expect the latter quantity to be minimized in the ground state. Honeycomb structures of trimesic acid molecules on a hexagonal lattice of sites have also been studied with Monte Carlo simulations.^49^ The simulations involved two short‐range pairwise interactions and lead to periodic superstructures with *N* up to 4 (lattice parameter ca. 4.5 nm).

Our model aims to describe the ground‐state structure of *C*_3_‐symmetric molecules exhibiting two dominating pairwise interactions characterized by the energies *ϵ*_Hc_ and *ϵ*_Hex_. The obtained ground‐state structure is the result of a competition between adsorption and interaction energies, and depends on the coverage *Θ*. We note that a given sample coverage, used as a global quantity, does not necessarily reflect a single (local) molecular density, but may be realized by a combination of areas with different molecular densities.

#### Assumptions

The interaction energy *E_N_* is defined as the average energy reduction of a single molecule owing to the interaction with neighboring molecules, while *ϵ*_Ads_ is the adsorption energy per molecule. *ϵ*_Ads_ is assumed to be constant for all molecules in all superstructures. Analogously, the two dominating pairwise interactions of the molecules are supposed to be independent of the order *N* of the superstructure. Adsorption on the second layer is assumed to be unfavorable because no second‐layer molecules were reported for the systems considered below.

We mainly focus on the case where the total energy of the molecules is dominated by adsorption rather than interaction energy, that is, |*ϵ*_Ads_|≫|*E_N_*|. This case is particularly interesting because 1) predictions of the ground‐state superstructure can be made without a precise knowledge of *ϵ*_Ads_ and 2) this condition is often fulfilled for largish molecules on metal surfaces. Indeed, interactions mediated by hydrogen bonds and dispersion forces bind with energies on the order of 0.1 eV, while adsorption of molecules is often much stronger, that is, |*ϵ*_Ads_| in the order of a few electron volts per molecule. In the present case of physisorbed Me‐TOTA calculations yielded |*ϵ*_Ads_| about 2 eV.^44^, ^45^

Finally, kinetic aspects are neglected.

#### Energy Considerations

Hereafter, negative interaction and adsorption energies indicate attraction. For |*ϵ*_Ads_|≫|*E_N_*|, every adsorbed molecule reduces the energy of the system and the ground state is obtained by first maximizing the number of adsorbed molecules and then minimizing the intermolecular interaction energy in a second step (Supporting Information, Section IV). If not all available molecules can be accommodated in any superstructure of order *N*, the ground state is the superstructure with maximal density *ρ_N_*. Otherwise, the ground state is found among those superstructures that can lead to a coverage *Θ* by minimizing the interaction energy.

In the following, we consider a single phase with a superstructure *N*. Separation into several phases is not expected as discussed in the Supporting Information, Section V. Below we first derive expressions for the molecular densities and interaction energies that are required for the total energy minimization.

#### Molecular Density and Interaction Energy

**Figure 3:**
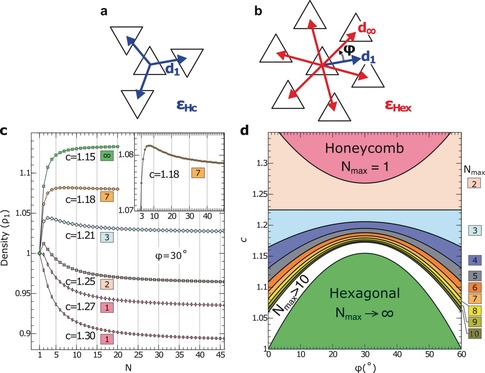
a) Honeycomb and b) hexagonal arrangements of *C*_3_ symmetric molecules represented by triangles. *d*_1_ (*d*_*∞*_) denotes the center‐to‐center distance between neighbor molecules in the honeycomb (hexagonal) stacking. *ϕ* is the angle between the vectors *d*_1_ and *d*_*∞*_ and can assume values between 0 and 60°. The pairwise interaction energies for the arrangements are a) *ϵ*_Hc_ and b) *ϵ*_Hex_. c) Evolution of the density (in units of *ρ*_1_) evaluated using Equation (2) as a function of the order *N* of the honeycomb superstructure. Curves were calculated for various *c*=*d*_1_/*d*_*∞*_ while keeping *ϕ*=30°. The numbers in colored boxes indicate the orders *N* of the superstructures that maximize the density for a given *c*. The inset is a zoom of the case *c=*1.18, which exhibits a maximum density for *N*=7. d) Order *N*_max_ of the superstructures with maximum density versus the distance ratio *c* and angle *ϕ*. The color code used for *N*_max_ is shown on the right. White color is used for *N*_max_>10.

Figure 3 a,b display representations of *C*_3_‐symmetric molecules in honeycomb and hexagonal arrangements. In the honeycomb mesh, every molecule has three nearest neighbors at a center‐to‐center distance *d_1_* (Figure 3 a). The number of nearest neighbors increases to six in the hexagonal arrangement (Figure 3 b), with a center‐to‐center distance *d*_*∞*_. The angle *ϕ* takes different stacking directions of the two configurations into account (Figure 3 b). With the above definitions, the unit‐cell area of a honeycomb superstructure of order *N* reads (Supporting Information, Section VI):

(1) $$A_{N}=\frac{\sqrt{3}}{2}\left\{3+c\left(N-1\right)\left[3\cos\varphi +\sqrt{3}\sin\varphi \right]+{\left(N-1\right)}^{2}c^{2}\right\}d_{1}^{2}$$

where *c*=*d*_1_/*d*_*∞*_. Since the number of molecules in the unit cell is *N_N_*=*N*(*N*+1), the molecular density is given by:

(2) $$\rho_{N}=\frac{N\left(N+1\right)}{A_{N}}$$

The densities *ρ*_1_ and *ρ*_∞_ of honeycomb *N*=1 and hexagonal structures are:

(3) $$\rho_{1}=\frac{4}{3\sqrt{3}d_{1}^{2}},\;\rho_{\infty }=\frac{2}{\sqrt{3}c^{2}d_{\infty }^{2}}$$

from which we find:

(4) $$\frac{\rho_{\infty }}{\rho_{1}}=\frac{3}{2}\frac{1}{c^{2}}$$

This implies that the molecular densities in the honeycomb *N*=1 and hexagonal structures are equal for *c*=$\sqrt{3/2}$ We note that for *c*=$\sqrt{3/2}\;$ , the largest molecular densities are achieved for *N*=2 and 3, and in particular *ρ*_2,_ *ρ*_3_>*ρ*_1_.

Different evolutions of the superstructures may be expected depending on the pairwise interaction energies. The interaction energies of the configurations depicted in Figure 3 a,b are *E*_1_=3 *ϵ*_Hc_ and *E*_*∞*_=6 *ϵ*_Hex_, where *ϵ*_Hc_ and *ϵ*_Hex_ are the energies of an edge–edge and a corner–edge bond, respectively. These values correspond to the energy reduction of a single Me‐TOTA molecule due to the interactions with neighboring molecules in honeycomb *N*=1 and hexagonal structures, respectively. The interaction energy in honeycomb superstructures of order *N* depends on the relative position of the molecules. Molecules at the edges of domains, at the corners, and in the hexagonal structure have interaction energies *ϵ*_Hc_+4 *ϵ*_Hex_, 2 *ϵ*_Hc_+2 *ϵ*_Hex_, and 6 *ϵ*_Hex_, respectively. In turn, the average interaction energy *E_N_* reads:

(5) $$E_{N}=6\frac{\left(N-1\right)\epsilon_{Hex}+\epsilon_{Hc}}{\;N+1}$$

where negative values of *E_N_*, *ϵ*_Hc_, and *ϵ*_Hex_ indicate attraction. The derivative of Equation (6)

(6) $$\frac{dE_{N}}{dN}=\frac{6}{{\left(N+1\right)}^{2}}\left(2\epsilon_{Hex}-\epsilon_{Hc}\right)$$

reveals that *E_N_* monotonously increases (decreases) with *N* for *ϵ*_Hc_/*ϵ*_Hex_>2 (*ϵ*_Hc_/*ϵ*_Hex_<2).

For low densities of Me‐TOTA on Au(111) the *N*=1 honeycomb structure is observed, which implies *E*_1_<*E*_*∞*_, and therefore *ϵ*_Hc_<2 *ϵ*_Hex_. Under this condition, *E_N_* increases with the order *N*, that is, high orders are unfavorable at low coverage. The discussion below pertains to this case, *ϵ*_Hc_/*ϵ*_Hex_>2.^50^

The evolution of the density *ρ_N_* with the order *N* of a honeycomb superstructure is shown in Figure 3 c for *ϕ*=30° and various ratios *c*. For *c*=1.30, the density decreases with *N* (rectangles in Figure 3 c). The *N*=1 structure consequently maximizes the density and minimizes the interaction energy making *N*=1 the ground state of the system for any coverage. The situation is different for *c=*1.15, where the molecular density continuously increases with *N* (green squares in Figure 3 c). The system evolves from a *N*=1 honeycomb lattice at low coverages into superstructures with larger *N* to accommodate further molecules at larger coverages. In practice, the maximum size *N* may be limited by kinetics and surface irregularities such as steps.

A markedly different evolution occurs for *c*=1.25. The density first increases from *N*=1 to *N*=2, and then continuously decreases towards larger *N* (crosses in Figure 3 c). Only superstructures with *N*=1 and 2 may be expected in this case. Larger *N* imply a less favorable interaction energy and also a reduced density. When the distance ratio *c* is changed, the density assumes a maximum at other values *N*=*N*_max_. For instance, *c*=1.18 leads to a maximum of *N*=7 (inset to Figure 3 c). We have calculated the maximal superstructure order *N*_max_ for a range of angles *ϕ* and distance ratios *c*. The results are displayed in Figure 3 d with colors representing *N*_max_. For any angle *ϕ*, any *N* may occur if the distance ratio *c* is in a suitable range.

#### Comparison of the Model to Experimental Data

The model was tested for a variety of superstructures of *C_3_* symmetric molecules. Table 1 summarizes the relevant parameters. System B corresponds to the present work. Systems C–I, K, and L were previously reported. A and J are fictitious cases with particularly small or large values of *c*.

**Table 1:** Parameters used in Figure 4 extracted from experimental observations (B‐I and K‐L) or fictitious (A, J). System B is Me‐TOTA.

System	*c*	*ϕ* [°]	*ϵ*_Hc_/*ϵ*_Hex_	$N_{max}^{theo}$	$N_{max}^{exp}$
A	0.60^[a]^	30^[a]^	2.50^[a]^	∞	
B	1.03	18.6	2.15^[b,c]^	∞	54^[e]^
C^23^, ^24^, ^26^	0.98	30	2.33^[d]^	∞	8^[e]^
D^29^	1.10	28.5	2.14^[b]^	∞	12^[e]^
E^27^	1.00	15	3.06	∞	8
F^28^	1.17	19.5	2.27	7	8
G^52^	1.17	48	2.73	5	5^[f]^
H^53^	1.50	26	2.83^[b]^	1	1^[e]^
I^30^	1.58	22	2.75^[a]^	2^[g]^	2^[g]^
J	2.00^[a]^	22	2.75^[a]^	1	
K^25^	1.19	35	1.14	5	2^[e]^
L^31^	1.16	57	1.76^[b]^	4	4^[f]^

[a] Fictitious value. [b] Calculated with the generalized amber force field.^51^, ^54^ [c] Upon substrate‐induced modification (Supporting Information, Section VII). [d] Extracted from Ref. 55. [e] Hexagonal structure was also observed. [f] Upon annealing the sample. [g] Only *N*>1 structures are based on the same pairwise interactions.

**Figure 4:**
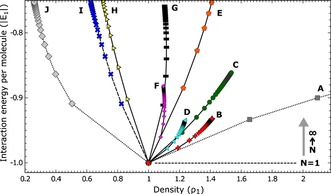
Parametric plot of the interaction energy per molecule [Eq. (5)] in units of |*E_1_*| versus molecular density [Eq. (2)] in units of *ρ*_1_ for honeycomb superstructures. Letters and symbols indicate different systems from Table 1. Lines connect the data points for each system starting from *N*=1 (point at coordinates (1,−1)) to *N*=2, 3,… (see inset on the right indicating the direction of increasing *N*). Upper ends of the curves correspond to *N*=∞. Negative interaction energies *E_N_* indicate attraction. For the case considered here, *ϵ*_Hc_/*ϵ*_Hex_>2, *E_N_* increases with *N*.

For systems A–J, we calculated the interaction energies *E_N_* (in units of |*E*_Hc_|) and the densities *ρ_N_* (in units of *ρ*_1_) for different values of *N* (Figure 4). The pairwise‐interaction energies were either extracted from the corresponding reference or calculated using the generalized AMBER force field.^51^ Owing to the normalization, the *N*=1 honeycomb structure has a density of 1 and an interaction energy per molecule of −1 in all cases. The interaction energy per molecule increases as the order *N* is increased, that is, structures with higher *N* are less favorable.

Superstructures of order *N* can be obtained through control of the surface coverage so long as the density increases with *N*. Our model predicts that this is the case for systems B–E and in the fictitious scenario A. Hexagonal lattices were indeed reported for B–D with the orders of the largest observed structures scattering between 7 and 54. These upper limits may have various reasons including limited control of the coverage, kinetics, and surface inhomogeneities. Systems D and E actually exhibit different superstructures at submonolayer coverages, which may be due to kinetics or a dependence of the adsorption energies on the superstructure. Furthermore, for the systems B and C, our model predicts a large number of high *N* superstructures within a small density interval. For instance, a coverage increase of Me‐TOTA by 0.6 % would change a *N*=50 superstructure into a hexagonal lattice. Consequently, small variations in densities between different sample areas lead to superstructures with different *N* as observed for Me‐TOTA and system C. It may be worth mentioning that between *N*=50 and *N*=∞, the interaction energy *E_N_* increases by only 0.3 % for Me‐TOTA, which may explain the larger number of defects in the *N*≈50 structures.

For systems H–J, the honeycomb lattice is preferred at all coverages because it minimizes the interaction energy and provides the most dense packing. Honeycomb superstructures up to *N*=4 were observed under special circumstances for system I. Motivated by this observation of only low *N*, we considered a fictitious ratio of *ϵ*_Hc_/*ϵ*_Hex_=2.75 that favors low order structures.^56^ System H exhibits both honeycomb and hexagonal lattices at submonolayer coverages suggesting that *ϵ*_Hc_≈2 *ϵ*_Hex_. For systems F, G, K, and L, the density initially increases and then decreases with *N*. In these examples the size of the honeycomb superstructures can be controlled up to *N*=*N*_max_. Our model predicts 5, 5, and 4 for systems F, G, K, and L, which is in line with the experimental observations (Table 1). Note that for K and L, *ϵ*_Hc_/*ϵ*_Hex_<2. For such cases, the molecules exhibit a hexagonal packing (*N*=∞) at low coverage, which can evolve into lower order (*N*<∞) honeycomb superstructures at larger coverage.

#### Case of a Reservoir of Molecules

The above model assumes 1)|*ϵ*_Ads_|≫|*E_N_*| and 2) a fixed number of available molecules. Condition (2) is violated when the system is coupled to a reservoir of molecules. This may for instance be the case when a concentrated solution of molecules is drop cast to the sample or when the molecular deposition is performed over a relatively long time. In this case the total binding energy for a superstructure *N* reads (Supporting Information, Section IV):

(7) $$E_{N}^{Tot}=\rho_{N}A\left(\epsilon_{Ads}+\frac{E_{N}}{2}\right)$$

where *A* is the surface area. Because both the adsorption *ϵ*_Ads_ and the interaction *E_N_* energies are assumed negative, it is favorable to accommodate as many molecules in the first layer as the density *ρ_N_* of the superstructure allows, i.e. *Θ*=*ρ_N_*. For |*ϵ*_Ads_|≫|*E_N_*|, Equation (7) simplifies to $E_{N}^{Tot}=\rho_{N}\epsilon_{Ads}$ , and the ground state of the system is the superstructure that maximizes the density *ρ_N_*. In contrast, for |*ϵ*_Ads_|≪|*E_N_*|, $E_{N}^{Tot}=\rho_{N}AE_{N}/2\rho_{N}E_{N}$ such that the ground state is the structure that minimizes *ρ_N_* *E_N_*. It may be worth mentioning that *ρ_N_* *E_N_* corresponds to the interaction energy density, that is, the quantity minimized by Xiao et al.^27^

For *ϵ*_Ads_≈*E_N_* the adsorption and interaction energies compete and the result is not straightforward. Ibenskas and Tornau^49^ derived a ground state phase diagram for this regime.

## Discussion

We recall that our model attempts to determine the ground state. Kinetic limitations may therefore lead to the observation of intermediate superstructures as illustrated by systems G and L (Table 1). The initial hexagonal and disordered metastable configurations evolve toward honeycomb superstructures of order *N*_max_ upon annealing. Trapping into metastable states may be facilitated when the energies involved are close to the ground state energy. This problem arises at large *N* where interaction energy differences are small. For instance the interaction energy differences between the *N*=50 and 51 structures are approximately 20 μeV for a single Me‐TOTA molecule and approximately 45 meV for the complete unit cell.

The model drastically simplifies the complexity of interactions and atomic positions at surfaces. It may nevertheless be useful beyond an interpretation of existing structures and provide some guidance for the design of molecules that implement certain superstructures. First, the decision between a honeycomb and a hexagonal lattice at low coverage is determined by the ratio *ϵ*_Hc_/*ϵ*_Hex_, that is, the respective strengths of the intermolecular attractions. Second, the geometric parameters *c*=*d*_1_/*d*_*∞*_ and *ϕ* may be adjusted to favor a particular lattice in the limit of large coverages (Figure 3 d). Finally, a large variation of the density with *N* (Figure 4) simplifies the control of the superstructure order *N* via the coverage. It also renders a superstructure more stable with respect to coverage variations.

## Conclusion

The triangular molecule Me‐TOTA forms honeycomb superstructures on Au(111) and Ag(111). The characteristic scale of the patterns is controlled by the molecular coverage. The largest unit cells observed (ca. 3000 molecules) are significantly larger than previously reported coverage‐controlled honeycomb structures.^23^, ^24^, ^25^, ^26^, ^27^, ^28^, ^29^, ^30^, ^31^ We developed a general three‐parameter model of the energetics of honeycomb superstructure of *C*_3_ symmetric molecules. The ground‐state structure is rationalized in terms of energy minimization rather than a surmised energy density optimization. The model reproduces important aspects of the present experimental results as well as several previously reported structures. This demonstrates the versatility of the model, which may in turn be used to guide the design of molecules for honeycomb superstructures.

## Conflict of interest

The authors declare no conflict of interest.

## Supporting information

As a service to our authors and readers, this journal provides supporting information supplied by the authors. Such materials are peer reviewed and may be re‐organized for online delivery, but are not copy‐edited or typeset. Technical support issues arising from supporting information (other than missing files) should be addressed to the authors.

Supplementary
