# Supplementary material for: Coverage‐Controlled Superstructures of C 3‐Symmetric Molecules: Honeycomb versus Hexagonal Tiling
Source: Angew Chem Int Ed Engl. 2020 Mar 20;59(18):7008–17. doi: 10.1002/anie.202001383 (PMC7216838; doi:10.1002/anie.202001383)
Supplement: Supplementary file 1 — Supplementary [file ANIE-59-7008-s001.pdf]

Angewandte Chemie
Eine Zeitschrift der Gesellschaft Deutscher Chemiker

Supporting Information

Coverage-Controlled Superstructures of C_3-Symmetric Molecules: Honeycomb versus Hexagonal Tiling

Torben Jasper-Tönnies, Manuel Gruber,* Sandra Ulrich, Rainer Herges, and Richard Berndt*

anie_202001383_sm_miscellaneous_information.pdf

I. EXPERIMENTAL SECTION

Synthesis: Me-TOTA molecules were synthesized following Ref. 1.
Sample preparation: Clean and flat Au(111) and Ag(111) surfaces were prepared by repeated cycles of Ar sputtering and subsequent annealing. Molecule deposition was performed by sublimation from a Ta crucible under pressures below 10^{-7} Pa onto a sample held at ambient temperature. The samples were then inserted into a cryogenic STM without breaking the vacuum.
STM: Images were recorded with a home-built instrument at 4.6 K.

II. ADDITIONAL DATA OF ME-TOTA ON AU(111)

Figure S1 shows an overview topograph of Me-TOTA on Au(111), which exhibits superstructures of different orders. The coexistence of several superstructures, with different molecular densities, suggests that kinetic effects have hindered the convergence to the ground state characterized by a single superstructure.

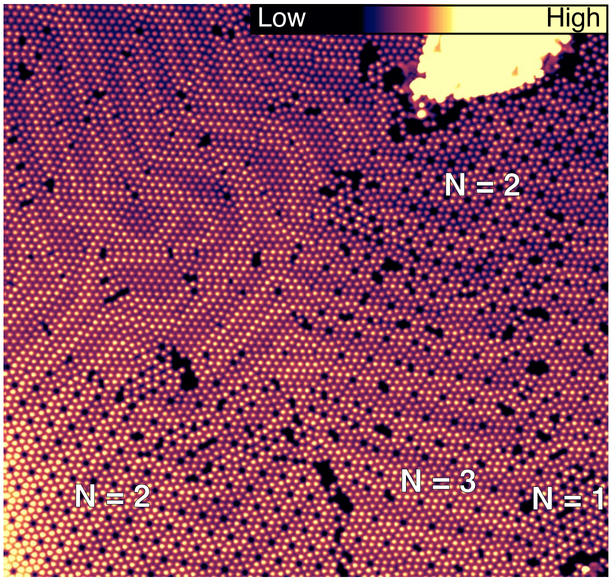

FIG. S1. Large-scale constant-current STM topograph (93 nm wide) of Me-TOTA on Au(111). Different areas of the sample are labeled with the order of the corresponding superstructure.

The dense area in the top-left part of the image (Figure S1) exhibits several defects, such as missing molecules and an apparent absence of regularity in the structure. The area is composed of fragments of superstructures where the orders range from $N \approx 10$ to ≈ 13. The absence of regularity is presumably due to the herringbone reconstruction (bright yellowish lines in Figure S1), which disturbs the epitaxial relationship between the molecules and the substrate.

As stated in the main text, the pairwise interactions between the molecules make the honeycomb superstructures chiral. In Figure S2, the enantiomer superstructures of order $N = 1$, 2 and 3 of those shown in Figure 1 of the manuscript are displayed.

III. ME-TOTA HONEYCOMB SUPERSTRUCTURE ON AG(111)

Comparing Figure S3 with that of Figure 1 of the main text, we find that the honeycomb superstructures observed on Ag(111) are essentially the same as on Au(111). In both cases, the molecules within a domain are arranged with a corner-to-side configuration (*e. g.*, molecules marked by magenta or yellow triangles in Fig. S3) and occupy the same type of hollow adsorption site. Molecules belonging to neighboring domains are rotated by 60° relative to each other. The side-by-side configurations at domain boundaries on Ag(111) (Fig. S3b) and Au(111) (Fig. 1f of main manuscript) are identical.

The large superstructures on Ag(111) are more prone to defects at the unit cell corners. Figure S3a shows an example: the domain at the lower right (magenta) is interrupted by a row of molecules with a different orientation (green). These molecules actually occupy bridge sites, a case we did not observe for superstructures of intermediate N. In addition, the pores are sometimes occupied by a molecule (molecule marked in grey in Fig. S3a).

IV. TOTAL BINDING ENERGY

We recall that every superstructure of order N is associated with a molecular density ρ_N and an averaged interaction energy E_N. On a sample of surface area A with a molecular coverage θ, θA molecules are available. To simplify the discussion, we only consider coverages θ that match given molecular density ρ_N. In this case, the ground state structure can be described with a single superstructure N, while intermediate coverages may involve two superstructures.

Every molecule adsorbed within the first layer reduces the total binding energy by ε_{Ads} (adsorption) and by $E_N/2$ (interaction with neighboring molecules), while other molecules (in the gas phase or second layer) do not contribute to the binding energy. For a superstructure N with $\rho_N \geq \theta$, *i. e.* a superstructure that can accommodate all available molecules, the total binding energy is:

$$E_N^{\text{Tot}} = \theta A \left(\varepsilon_{\text{Ads}} + \frac{E_N}{2} \right), \tag{S1}$$

whereby parts of the surface may remain molecule-free. However, if $\rho_N < \theta$, only $\rho_N A$ molecules are in the first layer, while $\theta A - \rho_N A$ molecules are in the second layer or in the gas phase and do not contribute to the binding

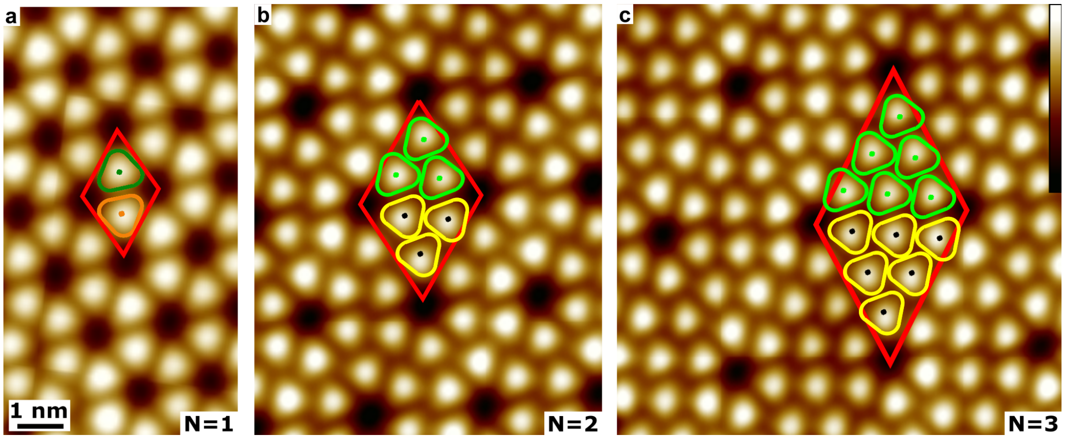

FIG. S2. (a–c) Constant-current STM topographs of a series of honeycomb superstructures of Me-TOTA on Au(111). Similarly to Figure 1 of the main text, some of the molecules are marked with rounded triangles whose color is representative of the molecular adsorption site and of the orientation of the molecule relative to the substrate. The red rhombi show the unit cells of the honeycomb superstructures of orders (a) $N = 1$, (b) 2 and (c) 3.

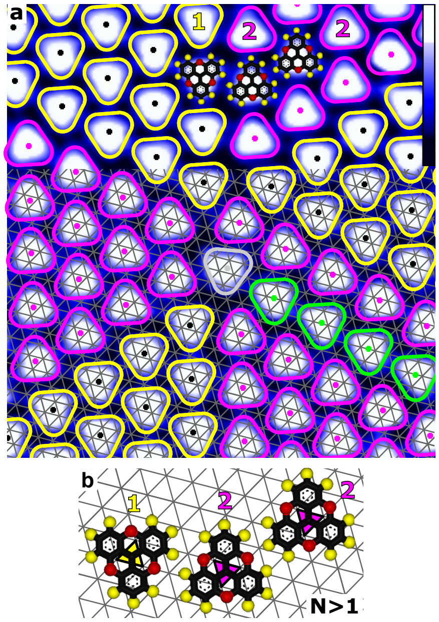

FIG. S3. (a) Constant-current STM topograph of Me-TOTA on Ag(111). The image show a a defect-rich area at the vertex of $N = 43$ unit cells, where the pore is occupied by a molecule (grey). A mesh is superimposed onto the lower part of the topograph to indicate the positions of the underlying silver atoms. The molecules marked in magenta and yellow are oriented as expected for the hexagonal domains. They also occupy different hollow adsorption sites. Green triangles point out molecules adsorbed at bridge sites forming a defect raw. Image width: 9.2 nm, tunneling parameters: 1 V, 30 pA. The inset shows the color scale used. (b) Ag lattice with scaled molecular structures illustrating the arrangement of molecules at domain boundaries. The pairwise geometries are identical to those on Au(111) (Figs. 1f and g of main manuscript). The numbers identify different adsorption sites (*e. g.*, 1 for *fcc* and 2 for *hcp*).

energy. In that case, the total binding energy reads:

$$E_N^{\text{Tot}} = \rho_N A \left(\varepsilon_{\text{Ads}} + \frac{E_N}{2} \right). \tag{S2}$$

Thus, the total binding energy of a structure and $\theta\, A$ available molecules can be expressed as:

$$E_N^{\text{Tot}} = \begin{cases} \theta A \left(\varepsilon_{\text{Ads}} + \frac{E_N}{2} \right), & \text{if } \rho_N \geq \theta. \\ \rho_N A \left(\varepsilon_{\text{Ads}} + \frac{E_N}{2} \right), & \text{otherwise.} \end{cases} \tag{S3}$$

The ground-state superstructure N is the one that minimizes Equation S3.

For $\varepsilon_{\text{Ads}} \gg E_N$, the interaction between the molecules may be viewed as a perturbation. In that case, the adsorption energy is minimized first, followed by a minimization of the interaction energy. The first minimization is realized by fulfilling $\rho_N \geq \theta$, as this condition ensures that all available molecules are adsorbed within the first layer. From the subset of superstructures N minimizing the adsorption energy, the ground state is the one that minimizes the interaction energy E_N. The resulting superstructure N is the one minimizing the total binding energy.

In the particular case that the system is coupled to a reservoir of molecules, the coverage is not fixed but follows the molecular density ρ_N, as every adsorbed molecule reduces the total binding energy, *i. e.* molecule-free areas on the sample are energetically unfavorable. The total binding energy then simplifies to:

$$E_N^{\text{Tot}} = \rho_N A \left(\varepsilon_{\text{Ads}} + \frac{E_N}{2} \right). \tag{S4}$$

In other word, the ground state for that case is the superstructure minimizing the energy density E_N^{Tot}/A.

V. SINGLE PHASE N VS. PHASE SEPARATION

Below we determine conditions under which a single phase being energetically preferred over several phases. We consider a surface area with coverage ρ_N and superstructure order N that decomposes into two phases of orders α and β covering fractions x_α and x_β of the area. This implies

$$\rho_N = x_\alpha \rho_\alpha + x_\beta \rho_\beta \quad \text{and} \quad x_\alpha + x_\beta = 1. \tag{S5}$$

Using Equations S5, the fraction x_α covered with phase α reads:

$$x_\alpha = \frac{\rho_N - \rho_\beta}{\rho_\alpha - \rho_\beta} \tag{S6}$$

We first consider the case $\varepsilon_{\text{Hc}}/\varepsilon_{\text{Hex}} > 2$. As shown in the manuscript, this condition leads to interaction energies E_N that increase with N. We further assume geometric parameters c and φ of the pairwise interactions (defined in Figs. 3a and b) that lie in the green area of Figure 3d. This assumption leads to *increasing* densities ρ_N with increasing N. The orders α and β then are lower and higher, respectively, than N (cf. Eq. S5). The relations between the interaction energies and the densities read

$$E_\alpha < E_N < E_\beta \quad \text{and} \quad \rho_\alpha < \rho_N < \rho_\beta. \tag{S7}$$

Neglecting energy contributions of phase boundaries and using Equations S5 the total interaction energy of the two phases is given by:

$$\frac{N_\alpha E_\alpha + N_\alpha E_\beta}{2} = \frac{x_\alpha A \rho_\alpha E_\alpha + (1 - x_\alpha) A \rho_\beta E_\beta}{2}, \tag{S8}$$

where N_α (N_β) is the number of molecules that arrange in phase α (β) and cover the area $x_\alpha A$ ($x_\beta A$). The factor 1/2 avoids double counting.

A single phase is preferred over several phases when[2]:

$$N_\alpha E_\alpha + N_\alpha E_\beta > N_A E_N. \tag{S9}$$

Using Equations S5, S6, S7, and S8 the above equation develops to

$$\frac{E_N - E_\beta}{\rho_N - \rho_\beta} > \frac{\rho_\alpha}{\rho_N} \frac{E_\beta - E_\alpha}{\rho_\beta - \rho_\alpha}. \tag{S10}$$

Since $\rho_\alpha/\rho < 1$, a more restrictive condition is

$$\frac{E_N - E_\beta}{\rho_N - \rho_\beta} > \frac{E_\beta - E_\alpha}{\rho_\beta - \rho_\alpha}. \tag{S11}$$

Figure S4 shows the interaction energy E_N as a function of coverage ρ_N for a fictitious system fulfilling Equation S7. The left and right terms of Equation S11 correspond to the slopes of the red and blue lines in Figure S4. Equation S11 is fulfilled in this case and generally when

$$d^2 E_N / d\rho_N^2 > 0. \tag{S12}$$

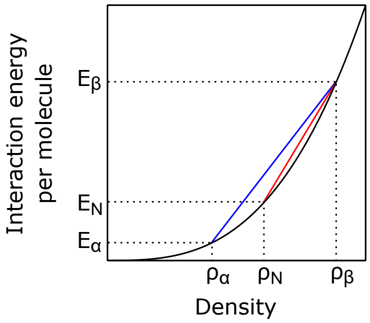

FIG. S4. Interaction energy E_N as a function of density ρ_N for a fictitious system satisfying Equation S11 . The red and blue lines exhibit slopes $(E_N - E_\beta)/(\rho_N - \rho_\beta)$ and $(E_\beta - E_\alpha)/(\rho_\beta - \rho_\alpha)$.

Systems B–E fall into this class.

A single phase is also expected for all systems that assume a maximal density at a finite order N_{max} (all other colors in Figure 1d) because their density increases up to this order and higher orders are inaccessible. This is the case of systems F and G.

Equation S12 is not satisfied for the fictitious system A. Nonetheless, a single phase is preferred because ρ_α/ρ_N is sufficiently small to fulfill Equation S10.

Finally, we address the case $\varepsilon_{\text{Hc}}/\varepsilon_{\text{Hex}} < 2$, *i. e.* systems in which E_N *decreases* with increasing N. Additionally we assume that ρ_N *decreases* with N, *i. e.* parameters c and φ that lie in the red area of Figure 3d. Arguments analogous to the ones used above apply and lead to the conclusion that a single phase is preferable. Systems K–L belong to this class with minimal density for the hexagonal structure ($N = \infty$) and a maximal density at a finite order N_{max}.

In summary, a single-phase ground state is perferred in all cases considered.

It may be worth noting that usually the number of molecules will be no integer multiple of the number of molecules per unit cell, $N(N+1)$. While this has a negligible effect on large terraces, it may become relevant when the molecules are confined to a small area.

VI. UNIT CELL AREA OF HONEYCOMB SUPERSTRUCTURES

A lattice vector $\vec{a}_{\text{I}}$ of the rhombic unit cell of a superstructure of order N is given by

$$\vec{a}_{\text{I}}(N) = \vec{d}_1 + \underline{R}(60°)\, \vec{d}_1 + (N-1)\, \vec{d}_\infty, \tag{S13}$$

where $\vec{d}_1$ and $\vec{d}_\infty$ are the lattice vectors of the simple $N = 1$ honeycomb and hexagonal meshes. $\underline{R}(\theta)$ is the matrix for an in-plane rotation by the angle θ. Equation S13 is illustrated in Figure S5 for $N = 3$.

The second lattice vector $\vec{a}_{\text{II}}$ of the superstructure reads:

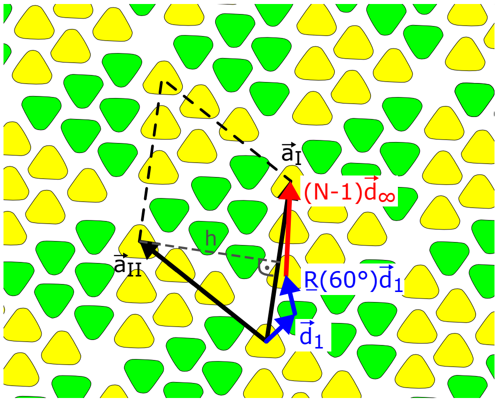

FIG. S5. Sketch of a $N = 3$ honeycomb superstructure. A rhombus (dashed black line) shows a unit cell with lattice vectors $\vec{a}_\mathrm{I}$ and $\vec{a}_\mathrm{II}$.

$$\vec{a}_\mathrm{II}(N) = \underline{R}(120^\circ)\,\vec{d}_1 + \underline{R}(60^\circ)\,\vec{d}_1 + (N-1)\,\underline{R}(60^\circ)\,\vec{d}_\infty \tag{S14}$$

$$= \underline{R}(60^\circ)\,\vec{a}_\mathrm{I}. \tag{S15}$$

The angle between $\vec{a}_\mathrm{I}$ and $\vec{a}_\mathrm{II}$ is therefore 60°, and $|\vec{a}_\mathrm{I}| = |\vec{a}_\mathrm{II}|$. The area A_N of the rhombus is

$$A_N = \frac{\sqrt{3}}{2}\,a_I^2(N). \tag{S16}$$

Considering the definitions of $\vec{d}_1$ and $\vec{d}_\infty$ (Figs. 3a,b of the main manuscript), we have:

$$\vec{d}_\infty = c\,\underline{R}(\varphi)\,\vec{d}_1,\;\; c = \frac{d_\infty}{d_1}. \tag{S17}$$

Equation 1 of the manuscript is obtained from Equation S16 using Equations S13 and S17.

VII. PAIRWISE INTERACTION ENERGIES OF ME-TOTA

The interactions energies were estimated from calculations with the generalized Amber force field[3] using Avogadro, an open-source molecular builder and visualization tool (Version 1.2.0).[4] The structure of the Me-TOTA molecules was fixed to that inferred from DFT calculations upon relaxation on Au(111).[1,5] The pairwise interaction energies $\varepsilon_\mathrm{Hc} = -160\,\mathrm{meV}$ and $\varepsilon_\mathrm{Hex} = -100\,\mathrm{meV}$ were obtained by minimization of the total energy of two molecules constrained to a plane.

Previous calculations predicted a charge transfer between the Me-TOTA molecules and the metal substrate.[1] This adds futher electrostatic interaction between the molecules. To estimate its energy, a partial charge $|q| = 0.3$ e (e: electron charge) was assumed to be localized to the center of the Me-TOTA molecule. Image charges in the substrate were also taken into account. This lead to a repulsive pairwise electrostatic interaction of $\approx 50\,\mathrm{meV}$. The total pairwise interaction energies are $\varepsilon_\mathrm{Hc} = -110\,\mathrm{meV}$ and $\varepsilon_\mathrm{Hex} = -50\,\mathrm{meV}$.

The pairwise interaction may in general be affected by the substrate, *e. g.* through deformation of the molecules. For the model presented in the manuscript, however, it is only necessary to determine whether $\varepsilon_\mathrm{Hc}/\varepsilon_\mathrm{Hex} > 2$ or < 2, *i. e.* whether the honeycomb or the hexagonal structure is more favorable. This information can be determined experimentally from measurements at submonolayer coverage.

Despite the above caveat, we observed that the ratios $\varepsilon_\mathrm{Hc}/\varepsilon_\mathrm{Hex}$ estimated from gas phase calculations are nonetheless consistent with the experimental observations for almost all systems in Table 1. The only exception is Me-TOTA, for which we found it necessary to take image charges into account.

[1] T. Jasper-Tönnies, I. Poltavsky, S. Ulrich, T. Moje, A. Tkatchenko, R. Herges, and R. Berndt, J. Chem. Phys. **149**, 244705 (2018).

[2] Negative energies indicate binding.

[3] J. Wang, R. M. Wolf, J. W. Caldwell, P. A. Kollman, and D. A. Case, J. Comput. Chem. **25**, 1157 (2004).

[4] M. D. Hanwell, D. E. Curtis, D. C. Lonie, T. Vandermeersch, E. Zurek, and G. R. Hutchison, J. Cheminform. **4** (2012), 10.1186/1758-2946-4-17.

[5] T. Jasper-Tönnies, A. Garcia-Lekue, T. Frederiksen, S. Ulrich, R. Herges, and R. Berndt, J. Phys.: Condens. Matter **31**, 18LT01 (2019).
